# Raloxifene Stimulates Estrogen Signaling to Protect Against Age- and Sex-Related Intervertebral Disc Degeneration in Mice

**DOI:** 10.3389/fbioe.2022.924918

**Published:** 2022-08-11

**Authors:** Neharika Bhadouria, Alycia G. Berman, Joseph M. Wallace, Nilsson Holguin

**Affiliations:** ^1^ School of Mechanical Engineering, Purdue University, West Lafayette, IN, United States; ^2^ Department of Mechanical and Energy Engineering, Indiana University-Purdue University Indianapolis, Indianapolis, IN, United States; ^3^ Weldon School of Biomedical Engineering, Purdue University, West Lafayette, IN, United States; ^4^ Department of Biomedical Engineering, Indiana University-Purdue University Indianapolis, Indianapolis, IN, United States; ^5^ Indiana Center of Musculoskeletal Health, Indianapolis, IN, United States; ^6^ Department of Orthopaedics, Icahn School of Medicine at Mount Sinai, New York, NY, United States

**Keywords:** hormone replacement/receptor modulators, menopause, aging, therapeutics, ovariectomy (OVX)

## Abstract

Estrogen agonist raloxifene is an FDA-approved treatment of osteoporosis in postmenopausal women, which may also be a promising prophylactic for painful intervertebral disc (IVD) degeneration. Here, we hypothesized that 1) aging and biological sex contribute to IVD degeneration by reducing estrogen signaling and that 2) raloxifene stimulates estrogen signaling to protect against age- and sex-related IVD degeneration in mice. 2.5-month-old (male and female) and 22.5-month-old (female) C57Bl/6J mice were subcutaneously injected with raloxifene hydrochloride 5x/week for 6 weeks (*n* = 7–9/grp). Next, female mice were ovariectomized (OVX) or sham operated at 4 months of age and tissues harvested at 6 months (*n* = 5–6/grp). Advanced aging and OVX increased IVD degeneration score, weakened IVD strength, reduced estrogen receptor-α (ER-α) protein expression, and increased neurotransmitter substance P (SP) expression. Similar to aging and compared with male IVDs, female IVDs were more degenerated, mechanically less viscoelastic, and expressed less ER-α protein, but unlike the effect induced by aging or OVX, IVD mechanical force was greater in females than in males. Therapeutically, systemic injection of raloxifene promoted ER-α protein to quell these dysregulations by enlarging IVD height, alleviating IVD degeneration score, increasing the strength and viscoelastic properties of the IVD, and reducing IVD cell expression of SP in young-adult and old female mice. Transcriptionally, injection of raloxifene upregulated the gene expression of *ER-α* and extracellular matrix-related anabolism in young-adult and old IVD. In vertebra, advanced aging and OVX reduced trabecular BV/TV, whereas injection of raloxifene increased trabecular BV/TV in young-adult and old female mice, but not in young-adult male mice. In vertebra, advanced aging, OVX, and biological sex (females > males) increased the number of SP-expressing osteocytes, whereas injection of raloxifene reduced the number of SP-expressing osteocytes in young-adult female and male mice and old female mice. Overall, injection of estrogen agonist raloxifene in mice normalized dysregulation of IVD structure, IVD mechanics, and pain-related SP expression in IVD cells and osteocytes induced by aging and biological sex. These data suggest that, in addition to bone loss, raloxifene may relieve painful IVD degeneration in postmenopausal women induced by advanced age, biological sex, and estrogen depletion.

## Introduction

Currently, there are no FDA-approved pharmacological treatments to prevent intervertebral disc (IVD) degeneration (Basso et al., 2017; Loibl et al., 2019), a major contributing factor to low back pain (Vasiliadis et al., 2014; Dowdell et al., 2017), or to restore IVD structure. The cost of healthcare and lost wages from low back pain exceeds 100 billion dollars annually in the United States (Katz, 2006). IVD degeneration is characterized by IVD height loss, extracellular matrix (ECM) breakdown, dehydration of the central nucleus pulposus, cell loss, and abnormal mechanical stresses (Zhao et al., 2007; Risbud et al., 2010; Homminga et al., 2012; Vincent et al., 2019). The hypovascularity and hypocellularity of the IVD challenge its self-repair and access to regenerative therapies (Gullbrand et al., 2018a). These intrinsic limitations have motivated the development of promising approaches to replace severely degenerated IVDs with tissue-engineered ones (Gullbrand et al., 2018b), but these invasive approaches that are applied at a late stage of IVD degeneration do not address the development of painful IVD degeneration and are not designed to spare patients the subsequent pain or cost of surgery. While low-level mechanical stimuli can protect the IVD (Holguin et al., 2009; Holguin et al., 2011; Holguin et al., 2013; Holguin and Silva, 2018), a pharmacological approach may be a more consistent regenerative therapy. Pharmacological agents administered systemically can reach the IVD (Holguin and Silva, 2018), and considering the potential relationship between osteoporosis and IVD degeneration (Livshits et al., 2010), treatments for bone structure may also target ECM-related pathways in the IVD.

Pre- and postmenopausal women develop greater IVD degeneration than age-matched men (Hoy et al., 2012; Wáng et al., 2016) and experience pain more frequently and at higher intensities (Vacca et al., 2014; Rosen et al., 2017). The use of raloxifene in women relieves self-reported back pain (Lyritis et al., 2010) and increases IVD height (Baron et al., 2005) for unclear reasons. Raloxifene hydrochloride is an FDA-approved non-uterine-targeting selective estrogen receptor modulator (SERM) that suppresses bone resorption by binding to estrogen receptors (ERs) in osteoclasts (Fuchs-Young et al., 1995; Taranta et al., 2002). The dramatic 50% reduction in vertebral fracture, which was previously unexplained by the mild 4% increase in bone mineral density (Ettinger et al., 1999), may also be associated with biophysical binding of water to collagen (Gallant et al., 2014) compared with ER signaling in bone (Bivi et al., 2016). Therefore, injection of raloxifene may be beneficial to the IVD by increasing water content and at least 2 other mechanisms:

(1) Raloxifene injection may regulate pain intensity via nociceptive processing in the central neural system (Papadokostakis et al., 2005). Discogenic pain by substance P (SP, neurokinin-1) has long been associated with IVD degeneration (Freemont et al., 1997). SP is a nerve signaling neurotransmitter and nociceptive pain marker that is negatively associated with ER-α protein expression in the IVD of postmenopausal women (Song et al., 2017). Considering that raloxifene binds ER-α (Goldstein et al., 2000), raloxifene may in turn reduce SP in the IVD.
(2) Raloxifene may engage Wnt signaling to promote the maintenance of notochordal cells and ECM of the IVD. The nucleus pulposus is the hydration core of the IVD, and healthy IVD is enriched with notochordal cells, rather than mature nucleus pulposus cells. Notochordal cells require Wnt signaling to maintain their cell phenotype (McCann and Séguin, 2016) and are better equipped than mature nucleus pulposus cells to produce ECM (Roberts et al., 2006). Aging and IVD degeneration perpetuate ECM degradation by reducing Wnt signaling (Holguin and Silva, 2018; Silva and Holguin, 2020), which triggers the replacement of notochordal cells by mature nucleus pulposus cells. Contrarily, the stabilization of Wnt signaling transcription factor β-catenin in the nucleus pulposus promotes notochordal cell proliferation, stimulates ECM anabolism, and protects the IVD from injury-induced ECM degradation (Holguin and Silva, 2018). Therefore, raloxifene may promote the proliferation of IVD cells (Gruber et al., 2002) by binding to ER-α (Goldstein et al., 2000) and potentiating Wnt/β-catenin signaling (Kouzmenko et al., 2004; Jia et al., 2016).

Here, we determined how aging, sex, and loss of estrogen affect IVD health. Further, we determined whether systemic injection of estrogen agonist raloxifene altered IVD structure, IVD mechanics, and the cellular expression of SP, ECM markers, and estrogen/Wnt signaling. Systemic drug delivery avoids iatrogenic IVD degeneration by local injection, but the effective quantity is dramatically reduced at the IVD because of its limited vascularization. Therefore, the pharmacological drugs must be potent, yet safe for repeated delivery. The FDA approved raloxifene for daily use in 1997, and it does not negatively engender back pain (Papadokostakis et al., 2005; Messalli and Scaffa, 2009).

To test the impact of repeated use, we injected raloxifene to C57Bl/6J mice 5x/week for 6 weeks. As expected, aging and OVX reduced trabecular BV/TV, whereas injection of raloxifene increased trabecular BV/TV in young-adult and old female mice. Similar to the consequences engendered by aging and/or biological sex, estrogen deficiency by ovariectomy induced mild IVD degeneration and increased pain-related neuropeptide SP, establishing that loss of estrogen may contribute to age-related IVD degeneration in female mice. By contrast, repeated injection of raloxifene augmented IVD structure and strength, diminished SP, and stimulated estrogen and Wnt signaling in young-adult IVD. In old mice, raloxifene similarly improved IVD structure and strength, reduced SP, and stimulated estrogen signaling but not Wnt signaling.

## Materials and Methods

### Mice

The objectives of this study were to investigate the effects of promoting estrogen by raloxifene in young and old IVDs and reducing estrogen by ovariectomy in mice. First, we determined the impact of biological sex on IVD structure and strength in response to raloxifene injection. Next, because raloxifene is administered clinically to aged women, we determined the effect of raloxifene injection in old female mice on IVD structure and strength. Lastly, to compare the IVD structure and strength changes induced by estrogen agonist raloxifene, we determined the effect of estrogen depletion on the IVD of young-adult mice. This *in vivo* study was approved by the Indiana University of School of Medicine Laboratory Animal Resource Center, Institutional Animal Care and Use Committee (IACUC). Tissues of the same set of mice were used in two different studies with different protocols.

Mice were housed in a 12-hour light/dark cycle and fed standard chow. In this study (Table 1), 4-month-old male and female (C57Bl/6J, *n* = 7–9/sex/group) mice served as the experimental control (CON), while another set of mice of both sex with a starting age of 2.5 months were injected with 0.5 mg/kg of raloxifene hydrochloride (SIGMA) for 6 weeks, 5x/week raloxifene (Ral), and tissues were harvested at 4 months of age. Next, 22.5-month-old female mice purchased from Jax (C57Bl/6J, *n* = 8/group) were injected with the same dose and frequency of raloxifene hydrochloride or vehicle PBS (VEH) and tissues were harvested at 24 months of age. Lastly, 4-month-old female mice (C57Bl/6J, *n* = 4–5/group) were ovariectomized and tissues were harvested at 6 months of age (OVX). Control mice were sham-operated (SHAM). Mice were euthanized by hypoxia as a primary means and by cervical dislocation as a secondary means. Lumbar and tail were harvested, and the spinal segments were divided up for specific testing (Table 2).

**TABLE 1:** Experimental design.

Cohort	Age (month)	Sex	Treatment/Surgery
Raloxifene	4	Female, Male	CON—control
			RAL—2.5-month-old mice injected with 0.5 mg/kg raloxifene hydrochloride for 6 weeks, 5x/week
	24	Female	VEH—vehicle PBS
			RAL—22.5-month-old female mice injected with 0.5 mg/kg raloxifene hydrochloride for 6 weeks, 5x/week
Ovariectomy	6	Female	SHAM—Sham-operated
			OVX—4-month-old female mice ovariectomized and harvested at 6 months of age

**TABLE 2:** Outcomes for each spinal level.

Outcome	Lumbar	Tail
Histology	L1-3	NA
qPCR (IVD)	L3-5	NA
Mechanics (IVD)	L6-S1	NA
µCT (vertebra)	L6	CC7

NA, not applicable.

### Histology and Immunohistochemistry

Motion segments were fixed in 15 ml of 10% formalin on a rocker for 24 h, submerged in 70% ethanol, embedded in paraffin, and sectioned (5 µm). The analysis included two mid-coronal sections for each sample. IVD morphology and histological score were determined from Safranin-O/Fast Green images on a scale of 14. In short, the nucleus pulposus (NP), annulus fibrosus (AF), and boundary between the two structures were scored based on structural properties. Normal NP (score = 0) consists of a single-cell mass with no visible cleft (Tam et al., 2018). Normal AF (score = 0) consists of a concentric laminar structure with no visible cleft. NP/AF boundary (score = 0) includes a clear demarcation between the NP and AF boundary. The addition of the NP, AF, and boundary score denoted the total score and connoted the degree of IVD degeneration. An increasing score indicates greater IVD degeneration, with a score of 0 as no degeneration and a score of 14 as the highest possible degeneration. Degeneration scoring was the average of 5 independent observers.

For IHC, samples were deparaffinized and rehydrated. Citrate buffer was used for 20 min at 90°C for epitope retrieval. Next, samples were incubated in PBS + BSA for 30 min at room temperature for blocking. Next, samples were incubated overnight at 4°C with primary antibody (ER (#MA3-310, Invitrogen): 1:200; SP (#ab14184, Abcam): 1:1500 and β-catenin (#9562S, Cell Signaling): 1:200). The secondary antibody Vectastain Elite ABC mouse IgG (kit PK-6102) was diluted in PBS + BSA at 1:200. ABC reagent was used as a vector based on the manufacturer’s protocol for 30 min. For ER-α (1 min) and SP (2 min), samples were submerged in 20 ml 0.05% DAB +0.01% H202 for chromagen development. 0.2% methyl green was used as a counterstain for 4-month-old samples. Lastly, the samples were dehydrated in gradations of ethanol and mounted with xylene. For quantification of protein expressions, the NP or AF was considered as regions of interest for protein intensity and cell count. The image was thresholded for intensity/cell count by semi-automation using ImageJ (NIH).

### Intervertebral Disc Mechanics

Based on a previous published technique (Liu et al., 2015), lumbar segment L6-S1 was harvested and the IVD was isolated by cutting through the growth plate of inferior and superior vertebrae using a dissection microscope (M400 Photomakroscop; Wild, Heerbrugg, Switzerland). Motion segments were imaged by X-ray (Bruker) and analyzed for IVD height using ImageJ (NIH) to calculate the deflection necessary to deliver a 5% strain. Further, IVDs were attached to a petri dish filled with phosphate-buffered saline (PBS, pH: 7.2) using cyanoacrylate glue to mimic the osmotic environment. IVDs were sinusoidally loaded using the micro-indentation system (Bio-Dent; Active Life Scientific, CA) for testing dynamic mechanical properties (Supplementary Figures S1A,A′). The motion segments were aligned with a 2.39-mm probe to cover the whole IVD and compressively strained to 5% at a frequency of 1 Hz for 20 cycles (preload of 18 g). The dynamic test was repeated in triplicate for each sample with 10 min of resting time between each trial.

Mechanical properties of the IVD were determined from the force–displacement curves (Supplementary Figure S1B) and included relative force (N), displacement (μm), loading stiffness (N/μm), energy dissipation (N·μm), and loss tangent. Relative force and displacement were calculated by considering the difference between the maximum and minimum values for these outcomes. Loading stiffness was calculated as the slope of the force relative to the displacement. Energy dissipation is a viscoelastic property that was calculated as the area under the hysteresis loop between the loading and unloading curves. The loss tangent is a measure of the extent of viscous over elastic nature of the tissue. The loss tangent represents the degree of viscoelasticity and was calculated as the phase shift between the force and displacement curves (Liu et al., 2015). Each value was the average of the two trials with the lowest standard deviation of three trials.

### Micro-Computed Tomography

Motion segments L6-S1 and CC6-7 were harvested and submerged in 1x PBS prior to imaging. Specimens were imaged using the Bruker SkyScan 1272 Micro-CT at a resolution of 8 μm. Images of the motion segment were contoured around the periosteal and the endosteal of the bone. For the trabecular analysis, the growth plate was used as a landmark and trabecular bone analysis consisted of the next 30 consecutive images (Silva and Holguin, 2020). For cortical analysis, the longitudinal center of the bone was identified, and 15 images above and below the centerline were analyzed using the Bruker CTan64 micro-CT software. The bone parameters measured include bone volume fraction (BV/TV), trabecular number (Tb.N), trabecular thickness (Tb.Th) for trabecular bone, and cross-sectional thickness (Ct.Th) and area (Ct.Ar) for cortical bone, using a lower threshold of 60 and upper threshold of 225 for analysis.

### qPCR

L3-5 IVDs were harvested, frozen in liquid nitrogen, pulverized, and suspended in TRIzol (#15596018, Invitrogen) until further processing (Caprez et al., 2018). RNA isolation and purification steps were followed (RNeasy Mini Kit, Qiagen), and RNA concentration was quantified (NanoDrop). cDNA was synthesized (iScript, Bio-Rad) from 400 ng of total RNA for the following TaqMan probes (Life Technologies): *Acan* (Mm00565794_m1), *β-catenin* (Mm01350387_g1), *Col1a1* (Mm00801666_g1), *Col2a1* (Mm01309565_m1), *ER-α* (Mm00433149_m1), *Foxa2* (Mm01976556_s1), *Tac1* (Mm04209856_m1), *Rela* (Mm00501346_m1), *Pi3k* (Mm01282781_m1), *Lef1* (Mm00550265_m1), *Lrp5* (Mm01227476_m1) and nerve growth factor (*NGF*) (Mm04209856_m1). Each gene expression was normalized to *18s* (Hs99999901_s1), and then, all deltaCT values were normalized to the average of the CON value using the 2^−ΔΔCT^ method.

### Statistics

Statistical analyses were performed using the Statistical Package for the Social Sciences (SPSS version 26) software. A one-way analysis of variance (ANOVA) was used to compare the effect of aging on female control IVD. If significant, the post hoc test was run to find the significant groups. With respect to ER-α and substance P immunohistochemistry, Student’s t-tests were used to compare 6-month-old with 24-month-old samples, since 4-month-old samples were counterstained with methyl green and direct comparisons with this group were not possible. In young-adult 4-month-old mice, a two-way ANOVA was used to compare the RAL with CON and to determine the main effects of treatment, sex, and their potential interaction in lumbar IVDs. Upon a significant interaction, a post hoc Tukey test was further conducted for 4-month-old IVDs. In old and OVX mice, Student’s t-test compared VEH with RAL groups or SHAM with OVX. Data are represented as box plots with mean marked as cross (x) and maximum/minimum whiskers or mean ± standard deviation. *P* < 0.05 was considered statistically significant.

## Results

### ER-*α* Protein Expression in Response to Advanced Aging, OVX, Raloxifene, and Raloxifene–Sex Interaction in the Intervertebral Disc

**FIGURE 1:**
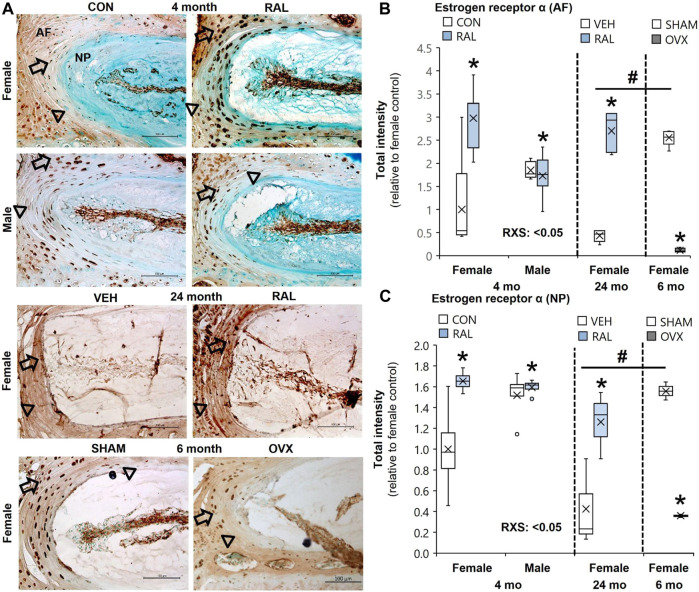
Raloxifene increased ER-α protein expression in young and old IVDs, whereas OVX reduced ER-α protein expression. **(A)** Estrogen receptor-α (ER-α) immunostaining (arrow: presence of stain, arrowhead: absence of stain) in the **(B)** AF and **(C)** NP of young-adult (4 months), old (24 months), and OVX (6 months) mice. Data are represented as box plots with mean marked as cross (x), 25/75% deviation lines, and maximum/minimum whiskers. *: control (CON, *n* = 5/sex/group) vs. raloxifene (RAL); R: CON vs. RAL; S: male vs. female; RxS: interaction, vehicle (VEH, *n* = 8/group) vs. RAL, SHAM (*n* = 4–5/group) vs. ovariectomized (OVX); ^#^: aging effect (4 months vs. 6 months vs. 24 months), *p* < 0.05. AF, annulus fibrosus; NP, nucleus pulposus. Scale: 100 μm.

Aging and OVX reduced ER-α protein intensity expression in the AF (Figures 1A,B) and NP (Figures 1A,C) compartments of the IVD (Supplementary Figures S2–S4). Compared with 6-month-old female SHAM IVDs, ER-α protein intensity expression in the AF (Figures 1A,B) and NP (Figures 1A,C) of 24-month-old female IVD was reduced by 85% (*p* < 0.01) and 72% (*p* < 0.05), respectively (Supplementary Figures S2–S4). Similarly, OVX reduced ER-α protein intensity expression in the AF and NP by 95% (*p* < 0.01) and 77% (*p* < 0.01), respectively (Figure 1; Supplementary Figures S2–S4). By contrast, raloxifene increased ER-α protein intensity expression in the AF (F = 8, *p* < 0.01; Figure 1B) and NP (F = 8, *p* < 0.01; Figure 1C) of the IVD of young-adult mice by 197% (*p* < 0.01) and 65% (*p* < 0.01), respectively (Supplementary Figures S2–S4). Old female mice responded similar to raloxifene injection by upregulating ER-α protein by 197% (*p* < 0.01) and 65% (*p* < 0.05) in the AF (Figure 1B) and NP (Figure 1C), respectively (Supplementary Figures S2–S4). While biological sex did not impact the protein expression of ER-α in the AF (Figure 1B) or NP (Figure 1C), raloxifene injection increased ER-α protein expression more in female IVD than in male IVD (interaction of raloxifene (R) x sex (S), F = 10, *p* < 0.01; Figures 1B,C, Supplementary Figures S2–S4). Positive ER-α cell counts showed a similar pattern of expression as protein intensity, which could also be affected by total cell loss or gain (Supplementary Figure S5). For 24-month female ER-α cell count, only protein intensity was included due to difficulty in counting cells in the aged IVDs (Supplementary Figure S5).

### Aging, Ovariectomy, and Biological Sex Increased Lumbar Intervertebral Disc Degeneration Score, but Raloxifene Reduced Lumbar Intervertebral Disc Degeneration Score and Augmented Intervertebral Disc Structure

**FIGURE 2:**
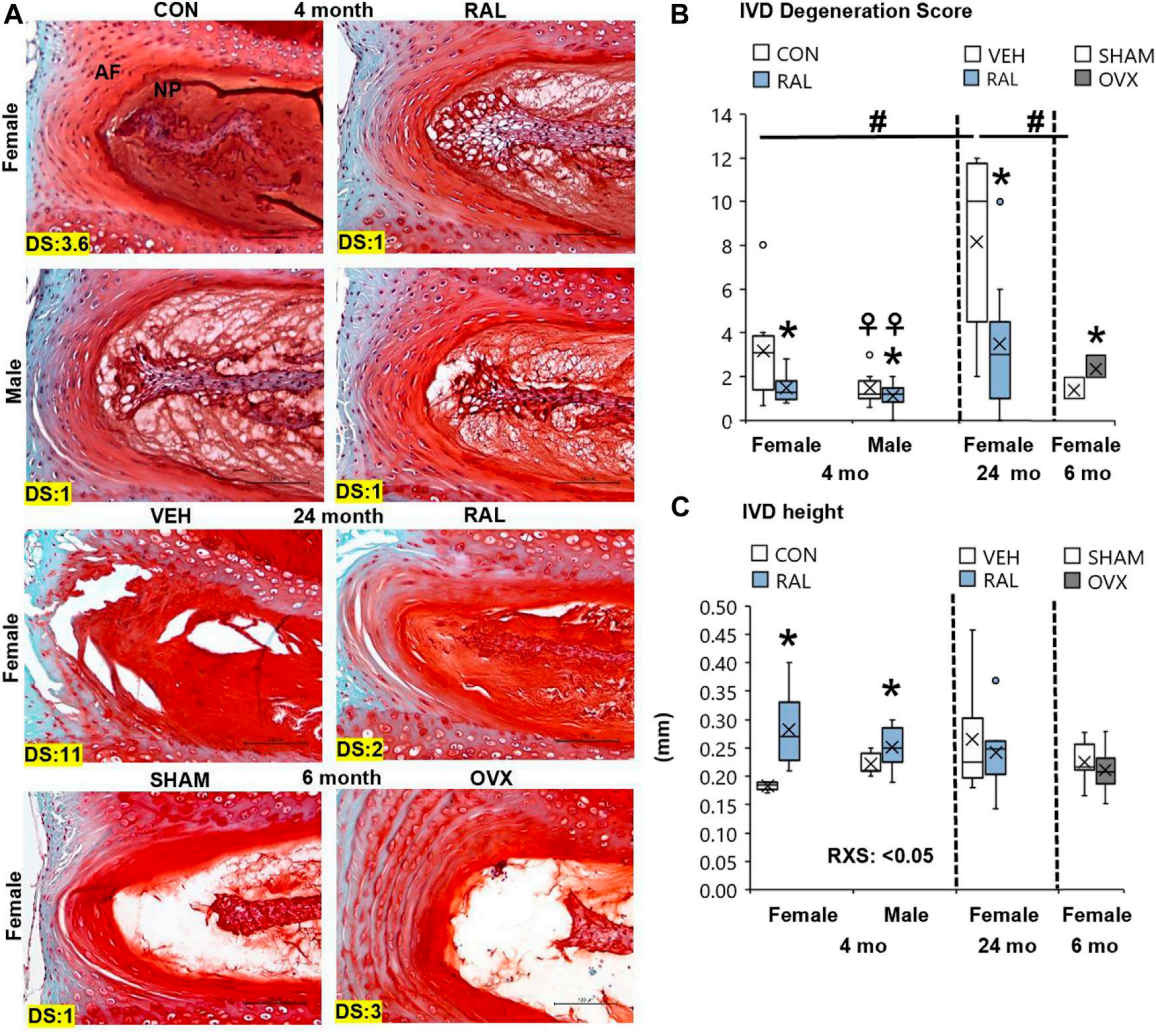
Raloxifene improved lumbar IVD degeneration score and IVD morphology. **(A)** 20x Safranin-O/Fast Green staining of IVD with disk degeneration score (DS) of each representative images bottom left corner. **(B)** IVD degeneration score, **(C)** IVD height of young-adult (4 months), old (24 months), and OVX (6 months) mice. Data are represented as box plots with mean marked as cross (x), 25/75% deviation lines, and maximum/minimum whiskers. *: Control (CON, *n* = 5/sex/group) vs. raloxifene (RAL); R: CON vs. RAL; S: male vs. female; RxS: interaction; vehicle (VEH, *n* = 8/group) vs. RAL, SHAM (*n* = 4–5/group) vs. ovariectomized (OVX); ^#^: aging effect (4 months vs. 6 months vs. 24 months); ^♀^: sex effect (male vs. female), *p* < 0.05. Scale: 100 μm.

Aging disorganized the NP and AF, reduced the NP cell band size, and increased the number of large, rounded inner AF cells (Figure 2A; Supplementary Figure S6). These morphological features demonstrated that 24-month-old female VEH IVDs were more degenerated (F = 7, *p* < 0.01) than 4-month-old CON IVD and 6-month-old SHAM disks by 137% (*p* < 0.05) and 146% (*p* < 0.01), respectively, based on IVD degeneration scoring (Figure 2B). Similarly, estrogen deficiency by OVX increased IVD degeneration score by 79% (*p* < 0.05, Figure 2B). Most of the histological structural changes in OVX occurred in the AF, with large spacing and proteoglycan staining between the lamellar fibers and large, round inner AF cells (Figure 2A). By biological sex, 4-month-old CON lumbar IVDs were more degenerated (F = 5, *p* < 0.05) in females than in males by 114% (*p* < 0.05) as demonstrated by greater NP disorganization, greater NP clefts, loss of NP cell band, and disorganized AF concentric bands (Figures 2A,C).

Preventatively, daily injection of raloxifene for 6 weeks reduced the lumbar sex-related IVD degeneration score (F = 4, *p* < 0.05) in young-adult female IVDs by 53% (*p* < 0.05) but no significant change (*p* = 0.12) in male IVDs (Figure 2B). Further, injection of raloxifene increased IVD height (F = 20, *p* < 0.01) by 55% (*p* < 0.01, Figure 2C) in female IVDs, with a greater IVD height benefit in female mice than in male mice, and increased nucleus pulposus area by 24% (F = 5, *p* < 0.05; Supplementary Figure S7). Compared with 24-month-old control IVD, injection of raloxifene reduced the lumbar age-related IVD degeneration score in old female mice by 57% (*p* < 0.05; Figure 2B) but did not impact the IVD height in old female mice (Supplementary Figure S7).

### Aging, Ovariectomy, and Biological Sex Impaired Lumbar Intervertebral Disc Compressive Force, but Raloxifene Strengthened Lumbar Intervertebral Disc Compressive Force

**FIGURE 3:**
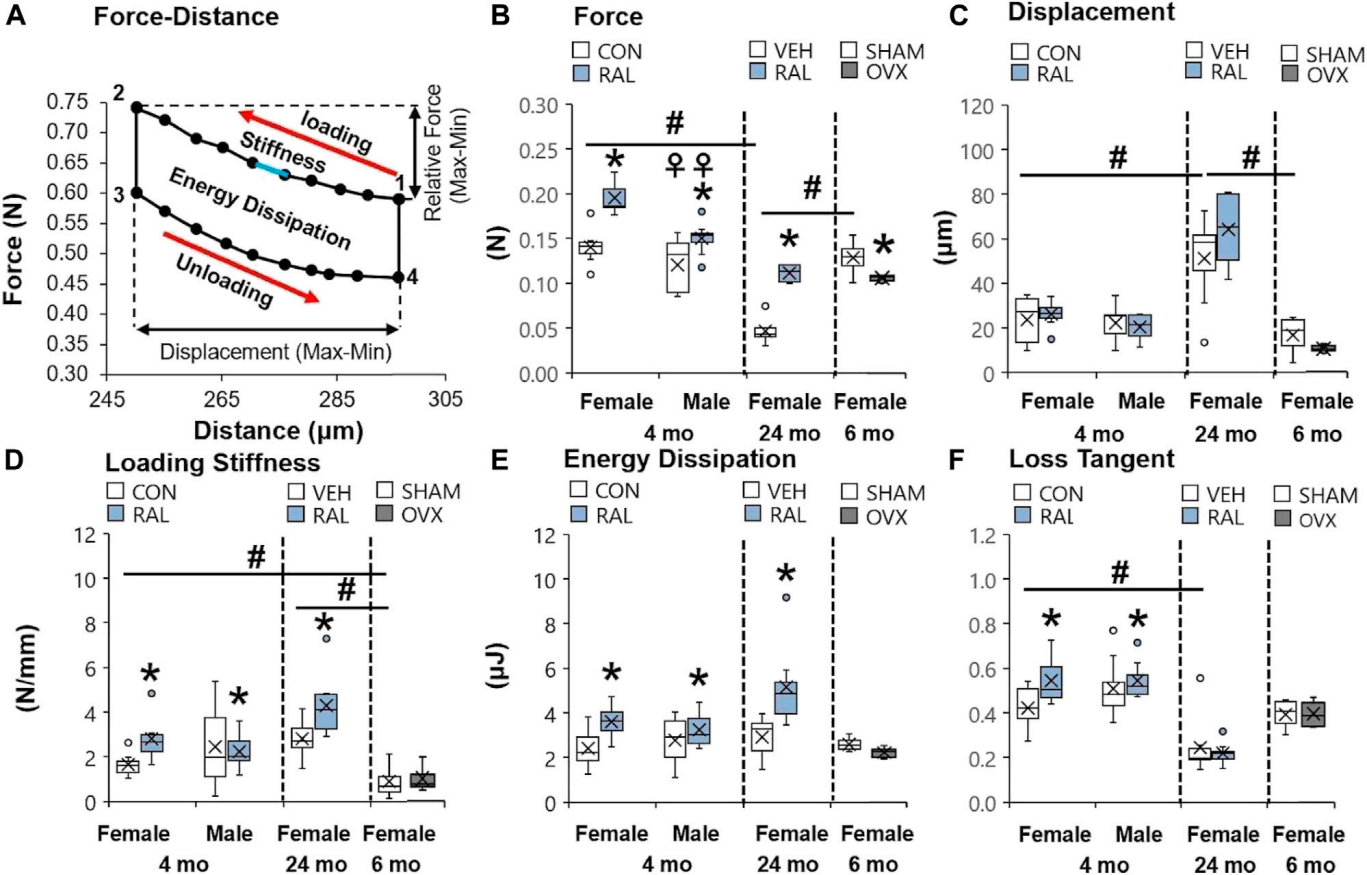
Raloxifene reversed the loss of strength and viscoelasticity in the IVD engendered by aging and biological sex. **(A)** Diagram of a single cycle of force–distance compressive loading of an IVD beginning at point 1 and ending at point 4. Mechanical properties included **(B)** relative force (2 minus 1 in y-axis), **(C)** displacement (2 minus 1 in x-axis), **(D)** loading stiffness (slope between 1 and 2), **(E)** energy dissipation (area inside the loops 1 through 4), and **(F)** loss tangent (phase shift between force and displacement curve). Data are represented as box plots with mean marked as cross (x), 25/75% deviation lines, and maximum/minimum whiskers. *: Control (CON, *n* = 5/sex/group) vs. raloxifene (RAL), SHAM (*n* = 4–5/group) vs. ovariectomized (OVX), vehicle (VEH, *n* = 8/group) vs. RAL; ^#^: aging effect (4 months vs. 6 months vs. 24 months); ^♀^: sex effect (male vs. female), *p* < 0.05.

Aging induced greater and more numerous impairments to the mechanical properties of the lumbar IVD than OVX or biological sex. Compared with 4-month-old and 6-month-old IVDs, advanced aging (24-month-old) reduced the compressive force of IVD (F = 33, *p* < 0.001) by 69% (*p* < 0.001) and 60% (*p* < 0.001; Figure 3B), respectively, and increased IVD displacement (F = 10, *p* < 0.01) by 115% (*p* < 0.01) and 206%, respectively (*p* < 0.01; Figure 3C). Similarly, compared with 4-month-old IVD, advanced aging increased the loading stiffness (F = 11, *p* < 0.01) by 42% (*p* < 0.01; Figure 3D) and reduced the loss tangent (F = 5.7, *p* < 0.01) by 50% (*p* < 0.01; Figure 3F). Depletion of estrogen in lumbar IVDs reduced the compressive force by 18% (*p* < 0.05; Figure 3B) and trended to reduce displacement (*p* = 0.09; Figure 3C) and energy dissipation (*p* = 0.08; Figure 3E) in IVDs. Compared with young-adult male IVDs, female IVDs resisted 17% (*p* < 0.01) more compressive force (Figure 3B).

Raloxifene injection increased the quasi-static and viscoelastic mechanical properties of young-adult and old IVD. In young-adult IVD, raloxifene injection increased IVD loading stiffness by increasing IVD compressive force by 122% (F = 27, *p* < 0.001; Figure 3B), but raloxifene injection did not alter IVD compressive displacement (F = 3, *p* = 0.09; Figure 3C). Similarly, raloxifene injection increased IVD viscoelastic properties such as energy dissipation and loss tangent by 32% (F = 6, *p* < 0.05; Figure 3E) and 17% (F = 4, *p* < 0.05; Figure 3F), respectively. Advanced aging did not impair the augmentation of quasi-static and viscoelastic mechanical properties of the IVD by injection of raloxifene, except for a lack of change in loss tangent (Figures 3B–F).

### Aging and Ovariectomy Increased Substance P Intensity in the AF, Whereas Raloxifene Reduced Substance P Intensity in Young-Adult and Old AF

**FIGURE 4:**
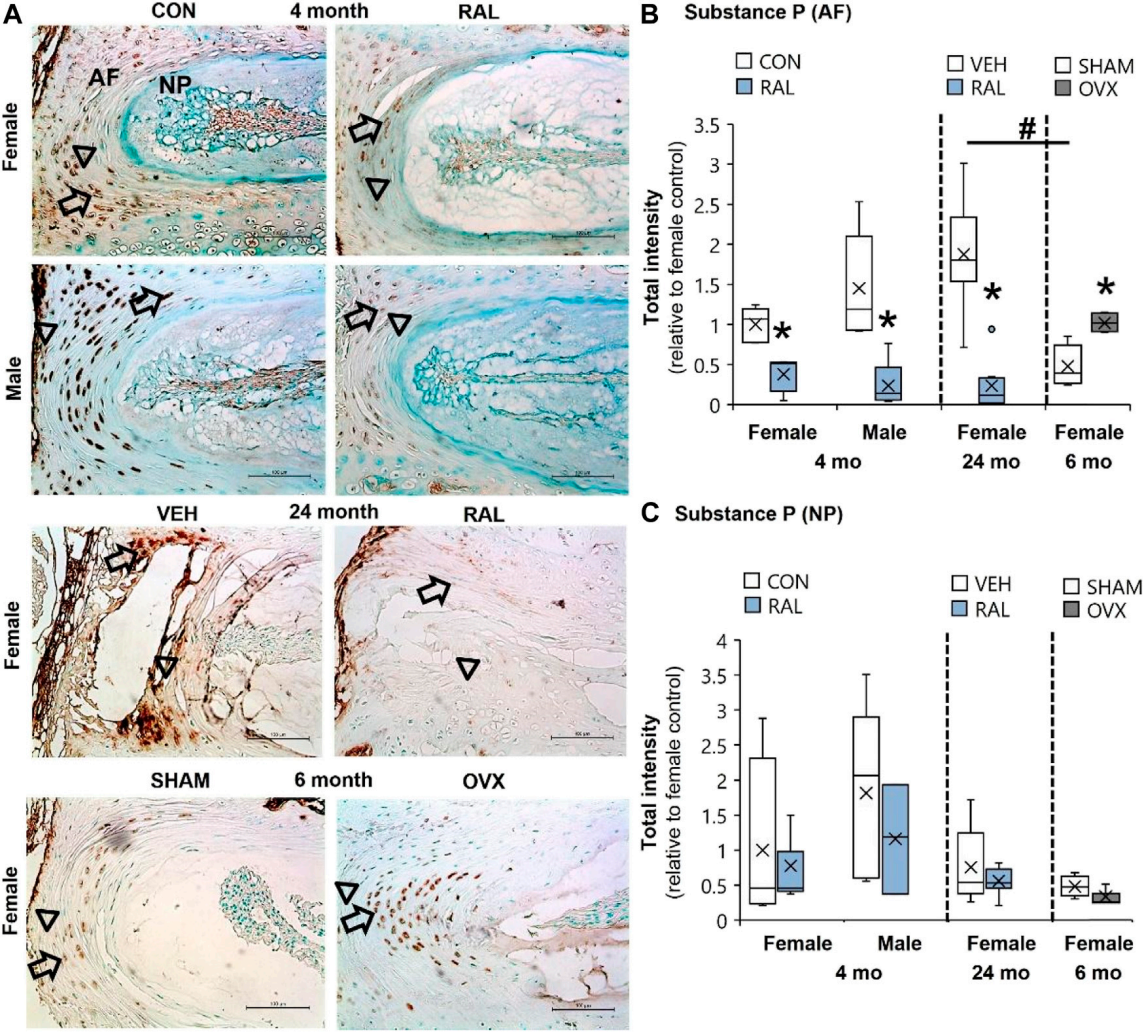
SP expression in the AF was elevated by aging and OVX and suppressed by raloxifene. **(A)** Substance P (SP) immunostaining (arrow: presence of stain, arrowhead: absence of stain) in the **(B)** AF and **(C)** NP of young-adult (4 months), OVX (6 months), and old (24 months) mice. Data are represented as box plots with mean marked as cross (x), 25/75% deviation lines, and maximum/minimum whiskers. *: Control (CON, *n* = 5/sex/group) vs. raloxifene (RAL), SHAM (*n* = 4–5/group) vs. ovariectomized (OVX), vehicle (VEH, *n* = 8/group) vs. RAL; ^#^: aging effect (4 months vs. 6 months vs. 24 months), *p* < 0.05. AF, annulus fibrosus; and NP, nucleus pulposus. Scale: 100 μm.

Compared with 6-month-old AFs, advanced aging increased SP expression by 290% (F = 25, *p* < 0.001) and OVX increased SP by 110% (*p* < 0.01; Figure 4B; Supplementary Figures S8–S10). By contrast, raloxifene injection reduced the SP expression (F = 26, *p* < 0.001) in the AF by 62% (*p* < 0.05) and 83% (*p* < 0.001) in 4-month-old females and males, respectively (Figure 4B). Similarly, raloxifene injection decreased the SP protein intensity by 87% (*p* < 0.001) in the AF of old female mice (Figure 4B). By contrast, neither aging, biological sex, OVX, nor raloxifene regulated SP expression in the NP (Figure 4C). The pattern of regulation was similar to cell count for SP staining, which could also be affected by total cell loss or gain (Supplementary Figure S11).

### Raloxifene Upregulated the Expression of ER-α, Transcription Factors, and ECM-Related Genes in Lumbar IVDs and Downregulated the Expression of Pain-Related Markers

**FIGURE 5:**
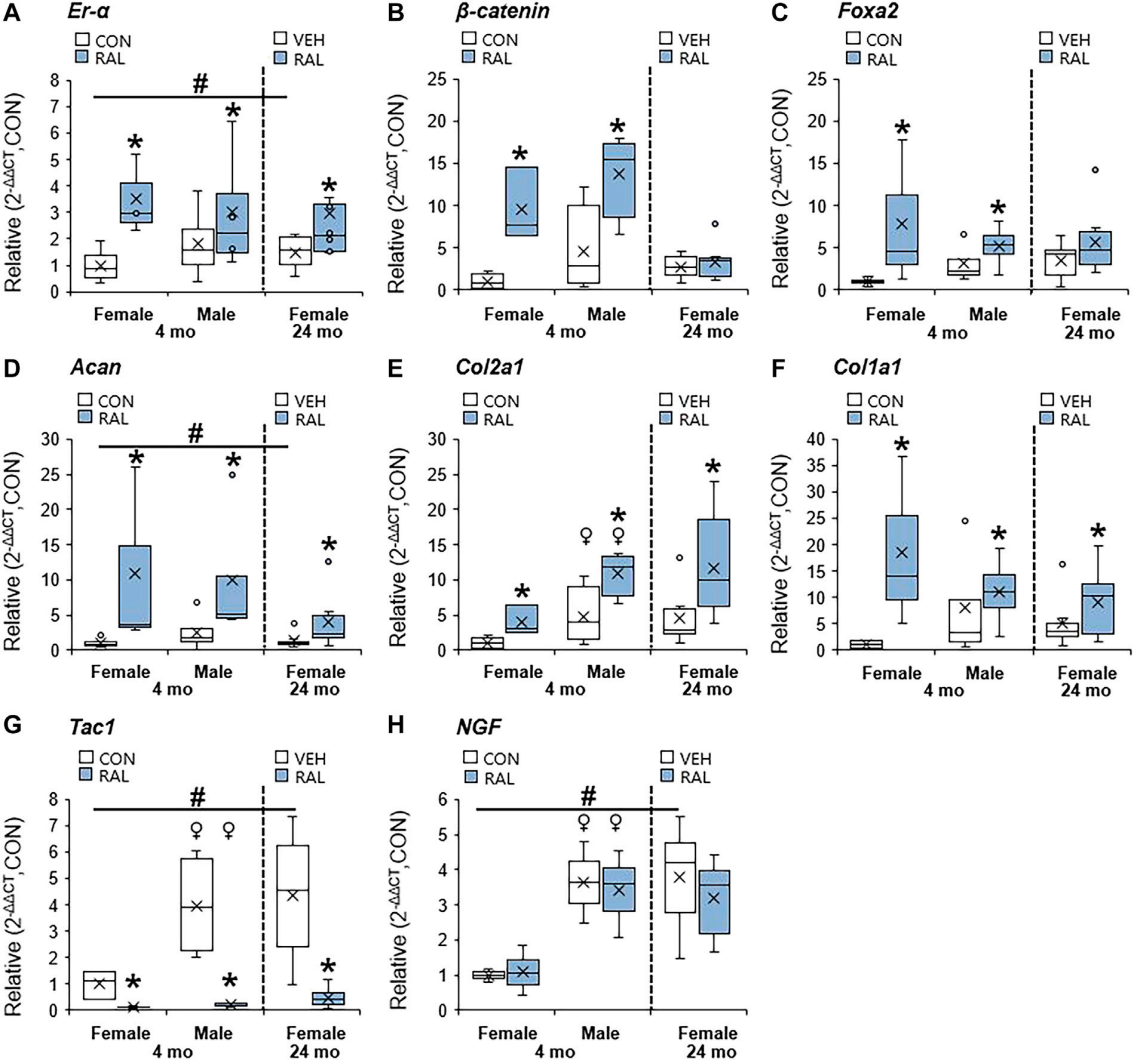
Raloxifene upregulated ER*-α,* transcription factors, and ECM-related expression, while suppressing the pain-related marker in young-adult and old lumbar IVDs. Gene expression of **(A)** *ER-α,* **(B,C)** transcription factors (*β-catenin*, *Foxa2*), **(D–F)** ECM (*Acan*, *Col2a1 and Col1a1*), and **(G,H)** nerve-related marker (*Tac1, NGF*) in the IVD. Data are represented as box plots with mean marked as cross (x), 25/75% deviation lines, and maximum/minimum whiskers. *: Control (CON, *n* = 5/sex/group) vs. raloxifene (RAL), vehicle (VEH, *n* = 8/group) vs. RAL; ^#^: aging effect (4 months vs. 6 months vs. 24 months); ^♀^: sex effect (male vs. female), *p* < 0.05.

**FIGURE 6:**
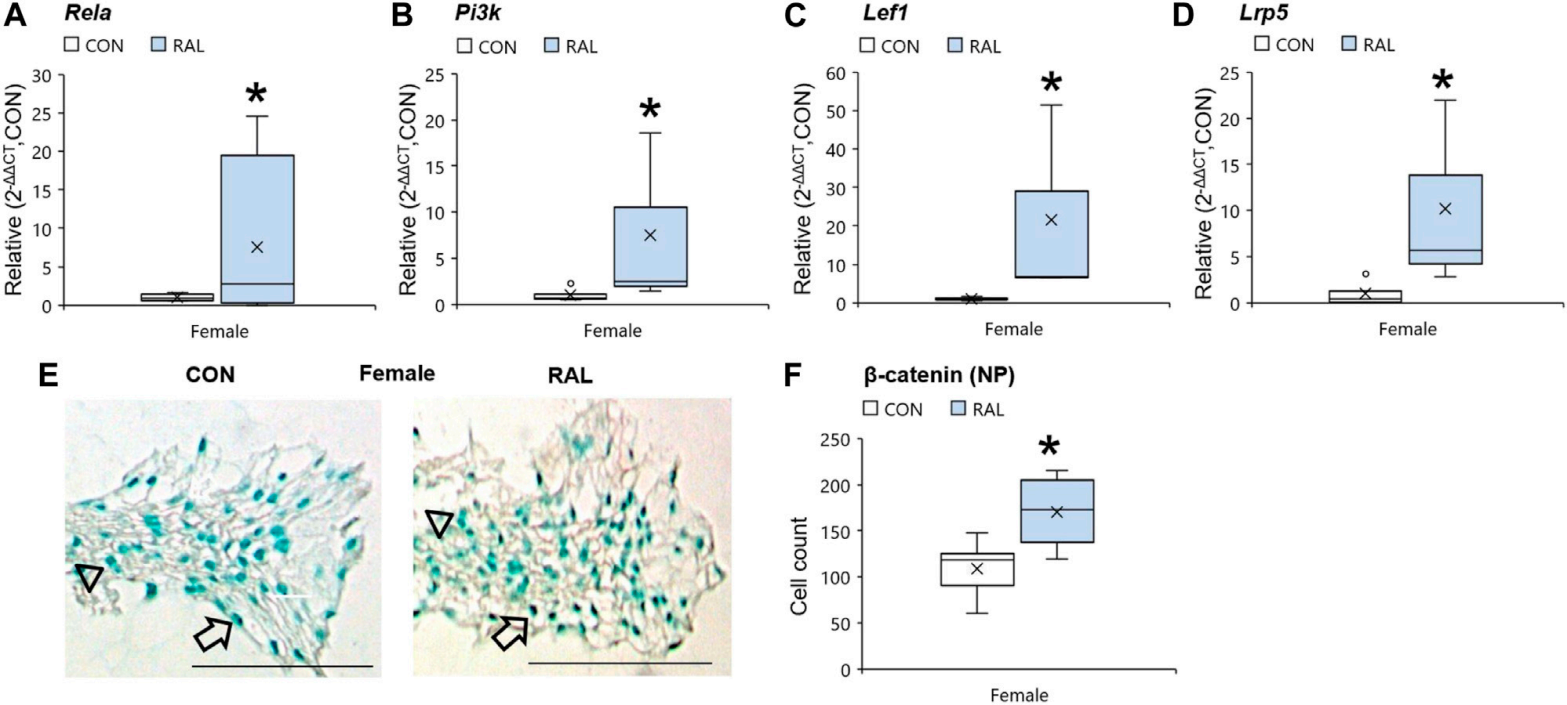
Raloxifene upregulated the gene expression of estrogen signaling and WNT signaling in the IVD. Gene expression of **(A)** *Rela,* **(B)** *Pi3k,* **(C)** *Lef1,* and **(D)** *Lrp5* in the IVD. β-catenin immunostaining (arrow: presence of stain, arrowhead: absence of stain) in the **(E)** NP of young-adult (4 months) female mice. Cell count of β-catenin protein expressing cells in the **(F)** NP of young-adult (4 months). Data are represented as box plots with mean marked as cross (x), 25/75% deviation lines, and maximum/minimum whiskers. *: Control (CON, *n* = 5/sex/group) vs. raloxifene (RAL). Scale: 100 µm.

Aging and biological sex induced mild changes to the expression of *ER-α,* (co-)transcription factors, and ECM-related and nerve-related genes compared with those engendered by raloxifene injection. Aging mildly upregulated the gene expression of *ER-α* and *Acan* by onefold (*p* < 0.05; Figures 5A,D) but strongly upregulated the gene expression of nerve-related markers *Tac1* (*p* < 0.001; Figure 5G) and *NGF* (*p* < 0.05; Figure 5H) by three-fold. Aging also upregulated the gene expression of *Col2a1* (Figure 5E) and *Col1a1* (Figure 5F) by four-fold to five-fold (*p* < 0.05). Compared with males, female IVDs expressed less *Col2a1* by five-fold (F = 9.1, *p* < 0.01; Figure 5E), which is suggestive of a switch in cellular phenotype. Female IVDs expressed less *Tac1* and *NGF* gene expression by five-fold (F = 9, *p* < 0.05; Figure 5G) and three-fold (F = 13, *p* < 0.01; Figure 5H), respectively. By contrast, raloxifene injection induced a robust transcriptional profile in young-adult and old IVD, possibly driven by the two-fold (F = 5, *p* < 0.05; Figure 5A) upregulation of *ER-α* because, while injection of raloxifene upregulated *β-catenin* (WNT signaling) and *FoxA2* (early NP cell marker) gene expression in young-adult IVD by 10-fold (F = 18, *p* < 0.01; Figure 5B) and eight-fold (F = 7, *p* < 0.05; Figure 5C), they were not regulated by raloxifene in old IVD. Moreover, raloxifene injection upregulated the expression of pro-anabolic ECM genes *Acan*, *Col2a1*, and *Col1a1* in young-adult mice by 11-fold (F = 9, *p* < 0.01; Figure 5D), four-fold (F = 10, *p* < 0.01; Figure 5E), and 18-fold (F = 9, *p* < 0.01; Figure 5F), respectively. In old female IVD, raloxifene injection upregulated the gene expression of *Acan*, *Col2a1,* and *Col1a1* by three-fold (*p* < 0.05; Figure 5D), three-fold (*p* < 0.01; Figure 5E), and two-fold (*p* < 0.05; Figure 5F), respectively. By contrast, raloxifene injection downregulated the gene expression of *Tac1* (F = 32, *p* < 0.001; Figure 5G) in female and male young-adult mice by eight-fold (*p* < 0.01) and 19-fold (*p* < 0.001), respectively. Likewise, raloxifene injection downregulated the gene expression of *Tac1* in old IVD by three-fold (*p* < 0.01; Figure 5G). Raloxifene injection did not regulate the gene expression of *NGF* in young-adult (F = 1, *p* = 0.57; Figure 5H) nor old (*p* = 0.1; Figure 5H) IVD. To further investigate the stimulation of estrogen signaling and Wnt signaling by injection of raloxifene, we determined that injection of raloxifene upregulated the gene expression of *Rela* and *Pi3K* by six-fold to eight-fold (*p* < 0.05) and *Lef1* and *Lrp5* by ninefold to 20-fold (*p* < 0.05; Figure 6). In addition, raloxifene increased the number of β-catenin–positive cells in the NP by 60% (Figure 6).

### Raloxifene Augmented Lumbar Vertebral Bone Structural Properties in Young and Old Mice

**FIGURE 7:**
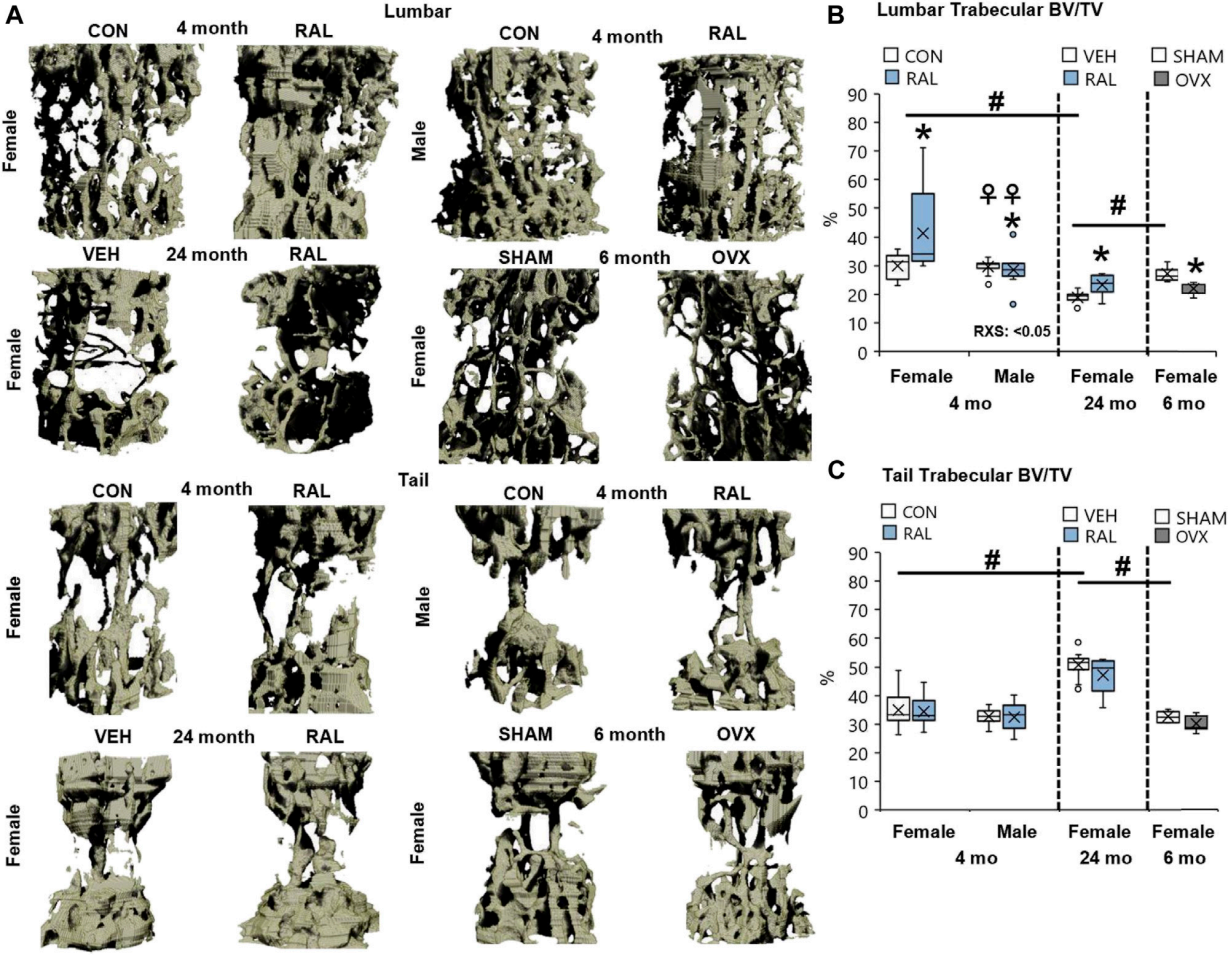
Raloxifene significantly increased the bone volume lost due to aging in lumbar vertebrae with no change in tail vertebrae. **(A)** 3D reconstruction of lumbar (L5) and tail vertebra (CC7) from young-adult (4 months), old (24 months), and OVX (6 months) mice. **(B)** Lumbar trabecular bone volume fraction (BV/TV), **(C)** tail trabecular bone volume fraction (BV/TV). Data are represented as box plots with mean marked as cross (x), 25/75% deviation lines, and maximum/minimum whiskers. *: Control (CON, *n* = 5/sex/group) vs. raloxifene (RAL); R: CON vs. RAL; S: male vs. female; RxS: interaction; vehicle (VEH, *n* = 8/group) vs. RAL, SHAM (*n* = 4–5/group) vs. ovariectomized (OVX); ^#^: aging effect (4 months vs. 6 months vs. 24 months); ^♀^: sex effect (male vs. female), *p* < 0.05.

Next, we corroborated the canonical regulation of lumbar vertebral structural properties by aging, biological sex, OVX, and raloxifene. Compared with 4-month-old young-adult and 6-month-old SHAM female mice, advanced aging reduced the lumbar trabecular bone volume fraction (Tb.BV/TV, F = 21, *p* < 0.001; Figures 6B, 7A) by 37% (*p* < 0.001) and 31% (*p* < 0.01), respectively. OVX reduced Tb.BV/TV by 17% (*p* < 0.05; Figure 7B) and Tb.N by 21% (*p* < 0.05; Table 3). By contrast, raloxifene injection increased Tb.BV/TV (F = 4, *p* = 0.02) in young-adult females by 38% (*p* < 0.05; Figure 7B) and trended to increase cortical thickness (F = 6, *p* < 0.05) by 10% (*p* = 0.10; Table 3). In old mice, raloxifene injection increased the Tb.BV/TV by 24% (*p* = 0.01; Figure 7B) but did not change cortical thickness (Table 3). More specifically, the structural adaptation of trabecular and cortical bone to estrogen agonist raloxifene was greater in females than in males as indicated by a significant interaction (Figure 7A; Table 3) and a qualitative increase in osteocytes that express ER-α (Supplementary Figure S12).

**TABLE 3:** Raloxifene augmented lumbar trabecular and cortical bone structure.

Spine level	Outcomes	4-month-old female		4-month-old male		24-month-old female		6-month-old female	
		CON	RAL	CON	RAL	VEH	RAL	SHAM	OVX
Lumbar	Tb.Th (mm)	0.06 ± 0.00^a^	0.07 ± 0.01	0.06 ± 0.00^b^	0.06 ± 0.01^b^	0.05 ± 0.00^a^	**0.06 ± 0.00** ^c^	0.05 ± 0.00^a^	0.05 ± 0.00
	Tb.N (1/mm)	4.9 ± 0.6^a^	5.8 ± 1.0	5.0 ± 0.34^b^	4.7 ± 0.87^b^	3.6 ± 0.48^a^	3.9 ± 0.59	3.1 ± 0.09^a^	**2.5 ± 0.44** ^c^
	Tb.Sp (mm)	0.06 ± 0.01^a^	**0.11 ± 0.03** ^c^	0.12 ± 0.01^b^	0.13 ± 0.02^b^	0.17 ± 0.01^a^	0.16 ± 0.02	0.13 ± 0.01^a^	0.14 ± 0.01
	Ct.Th (mm)	0.08 ± 0.01	**0.09 ± 0.01** ^c^	0.08 ± 0.01	**0.08 ± 0.01** ^c^	0.07 ± 0.01	0.08 ± 0.00	0.07 ± 0.01	0.07 ± 0.00
	Tt.Ar (mm^2^)	0.13 ± 0.04	0.14 ± 0.04	0.18 ± 0.02	0.18 ± 0.03	0.14 ± 0.02	0.14 ± 0.01	0.11 ± 0.02	0.12 ± 0.01
	Ct.Ar (mm^2^)	0.06 ± 0.01	0.07 ± 0.01	0.06 ± 0.01	0.06 ± 0.02	0.08 ± 0.01	0.08 ± 0.01	0.07 ± 0.01	0.07 ± 0.00
	Ct.Ar/Tt.Ar (%)	49.1 ± 0.11^a^	**54.0 ± 0.17** ^c^	31.0 ± 0.05^b^	34.1 ± 0.11^b^	53.0 ± 0.05^a^	**61.1 ± 0.04** ^c^	66.0 ± 0.05^a^	**57.0 ± 0.06** ^c^
Tail	Tb.Th (mm)	0.08 ± 0.01^a^	0.08 ± 0.01	0.08 ± 0.00^b^	0.08 ± 0.01^b^	0.11 ± 0.11^a^	0.10 ± 0.10	0.07 ± 0.0^a^	0.07 ± 0.0
	Tb.N (1/mm)	4.1 ± 0.5^a^	4.2 ± 0.5	4.3 ± 0.5	4.2 ± 0.5	4.7 ± 4.8^a^	4.6 ± 4.6	4.5 ± 0.3^a^	4.5 ± 0.4
	Tb.Sp (mm)	0.18 ± 0.01	0.19 ± 0.01	0.17 ± 0.01	0.17 ± 0.02	0.17 ± 0.02	0.17 ± 0.01	0.17 ± 0.01	0.17 ± 0.01
	Ct.Th (mm)	0.17 ± 0.01^a^	0.18 ± 0.02	0.17 ± 0.01	0.16 ± 0.02	0.22 ± 0.01^a^	0.21 ± 0.01	0.16 ± 0.01^a^	0.13 ± 0.03
	Tt.Ar (mm^2^)	0.20 ± 0.02^a^	0.21 ± 0.02	0.22 ± 0.02	0.20 ± 0.02	0.36 ± 0.03^a^	0.34 ± 0.05	0.19 ± 0.03^a^	0.16 ± 0.04
	Ct.Ar (mm^2^)	0.17 ± 0.01^a^	0.17 ± 0.02	0.17 ± 0.01	0.17 ± 0.03	0.24 ± 0.02^a^	0.23 ± 0.01	0.16 ± 0.02^a^	0.13 ± 0.03
	Ct.Ar/Tt.Ar (%)	79.0 ± 0.02^a^	79 ± 0.04	78.0 ± 0.04	79.1 ± 0.03	67.0 ± 0.02^a^	68.0 ± 0.11	76.1 ± 0.03^a^	72.0 ± 0.06

a Aging effect (4 months vs. 6 months vs. 24 months).

b Sex effect (female 4 months vs. male 4 months).

c Treatment effect (CON, *n* = 8–9/sex/group) vs. raloxifene-treated (RAL), vehicle (VEH, *n* = 8/group) vs. RAL, SHAM (*n* = 4–5/sex/group) vs. ovariectomized (OVX), *p* < 0.05. All data are average ±SD.

Bolding refers to a significant main effect of treatment.

Advanced aging increased Tb.BV/TV, Tb.Th, and Tb.N of proximal tail vertebrae by 56% (F = 21, *p* < 0.001; Figure 7C), 27% (F = 32, *p* < 0.001; Table 3), and 15% (F = 6, *p* < 0.01; Table 3), respectively, and increased cortical bone thickness by 23% (F = 41, *p* < 0.001; Table 3). These changes may not be reflected at the mid-vertebra. However, neither biological sex, OVX, nor raloxifene altered the structural properties of tail vertebrae (Table 3).

### Aging, Biological Sex (Females > Males), and Ovariectomy Increased the Number of Lumbar Vertebral Osteocytes Expressing SP, Whereas Raloxifene Reduced the Number of SP-Positive Osteocytes in Young-Adult and Old Mice

**FIGURE 8:**
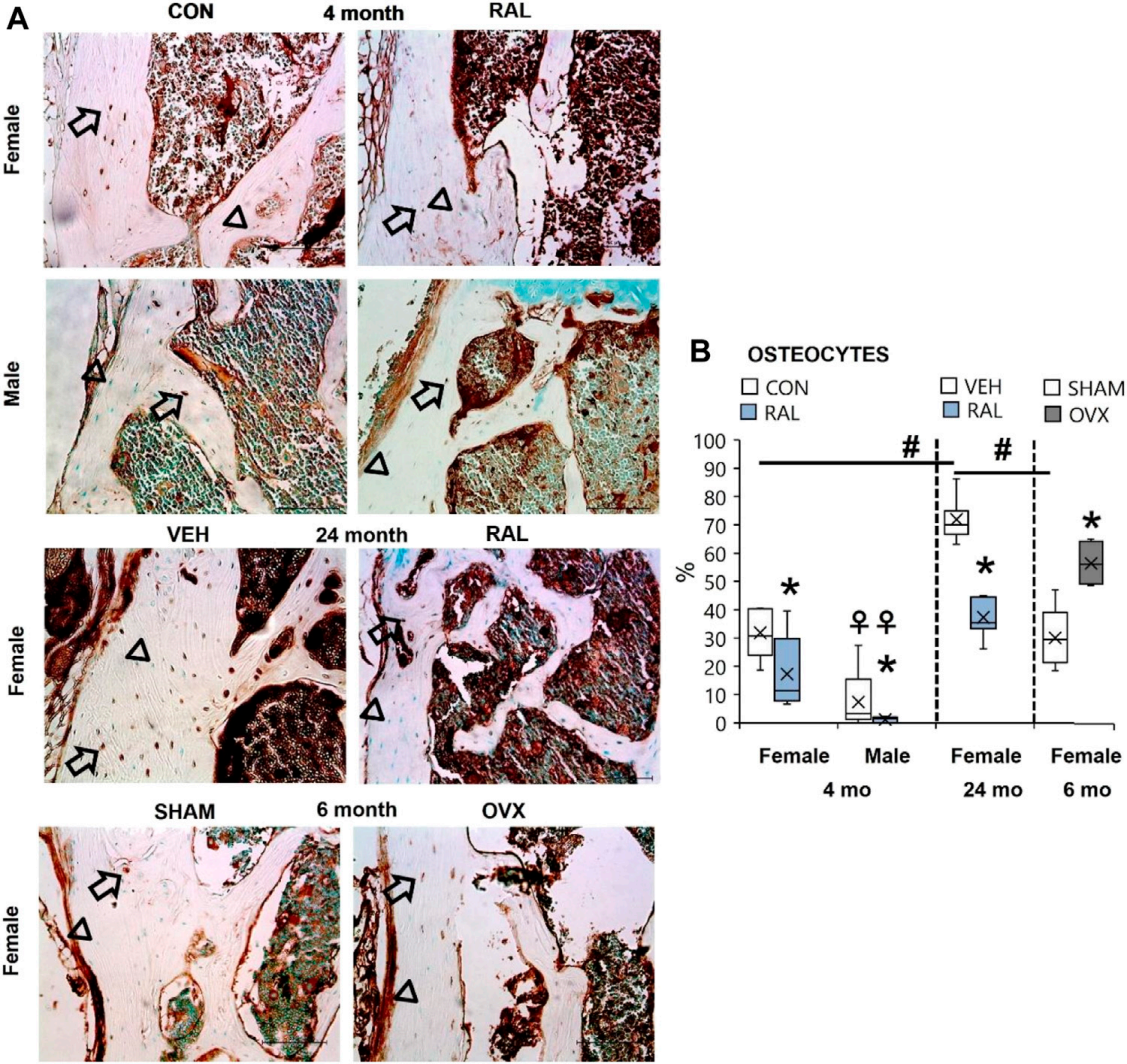
Age, biological sex, OVX, and raloxifene regulated the number of substance P-expressing osteocytes in lumbar vertebrae. **(A)** Substance P immunohistochemical staining (arrow: presence of stain; arrowhead: absence of stain) of young-adult (4 months), OVX (6 months), and old (24 months) mice. **(B)** Percentage of substance P-expressing osteocytes in L6 vertebra. Data are represented as box plots with mean marked as cross (x), 25/75% deviation lines, and maximum/minimum whiskers. *: Control (CON, *n* = 5/sex/group) vs. raloxifene (RAL); R: CON vs. RAL; S: male vs. female; RxS: interaction; vehicle (VEH, *n* = 8/group) vs. RAL, SHAM (*n* = 4–5/group) vs. ovariectomized (OVX); ^#^: aging effect (4 months vs. 6 months vs. 24 months); ^♀^: sex effect (male vs. female), *p* < 0.05. Scale: 100 μm.

Bone accrual requires innervation, and spinal pain may possibly be derived from bone signaling. Therefore, we interrogated the expression of substance P in osteocytes, as we have done so previously (Kroon et al., 2022). Advanced aging increased the percentage of SP-expressing osteocytes in old female mice by 40% (F = 43, *p* < 0.001) compared with young-adult female lumbar vertebrae (Figures 8A,B; Supplementary Figure S13). Similar effects were seen in aged female vertebrae compared with those in SHAM female lumbar vertebra. 4-month-old females have 18% (F = 21, *p* < 0.001; Figures 8A,B; Supplementary Figure S13) more SP-expressing osteocytes than males of the same age. Loss of estrogen in OVX increased the number of SP-positive osteocytes by 28% (*p* = 0.003; Figures 8A,B; Supplementary Figure S13). Similarly, young-adult female osteocytes expressed 335% (F = 10, *p* < 0.001; Figures 8A,B; Supplementary Figure S13) more SP-positive osteocytes than male osteocytes at the same age. By contrast, raloxifene injection reduced the number of osteocytes that expressed SP in young-adult and old mice by 54% (F = 7, *p* = 0.01; Figures 8A,B; Supplementary Figure 13) and 48% (*p* < 0.001; Figures 8A,B; Supplementary Figure 13), respectively.

## Discussion

We determined 1) the extent to which age, biological sex, and estrogen depletion induced features of IVD degeneration and 2) whether raloxifene injection could protect against these features in young-adult and old female mice. Advanced aging, biological sex (females relative to males) at a young-adult age, and OVX impaired IVD structure and strength, reduced the protein expression of ER-α, and increased the expression of neurotransmitter SP. Raloxifene injection prevents osteoporosis-related bone fracture in postmenopausal women by suppressing osteoclast resorption via binding of estrogen receptors. Here, subcutaneous injection of raloxifene attenuated age- and sex-related IVD degeneration, augmented IVD structural properties, and strengthened IVD mechanical properties by promoting extracellular matrix transcription. In IVD cells and osteocytes, raloxifene reduced the protein expression of SP in young-adult and old mice. In addition to the widely recognized ability of raloxifene to promote bone accrual, these data show that systemic administration of raloxifene may prevent and/or rescue critical IVD features lost with IVD degeneration and show great promise at potentially reducing discogenic back pain.

Maintenance of estrogen signaling in females may protect the IVD from age-related IVD degeneration. Postmenopausal women incur greater IVD degeneration than men (Wang, 2015), such as narrower IVD height (De Schepper et al., 2010), and incur greater IVD height loss than perimenopausal women, suggesting that estrogen deficiency may play a key role in IVD degeneration in women (Gambacciani et al., 2007). In rodents, signs of IVD degeneration as indicated by cell loss can begin as early as 2 weeks of age (Dahia et al., 2009), and while IVD-related sexual dimorphisms are not definitive in the literature (Vincent et al., 2019), young-adult female IVDs have been noted to be mechanically weaker than male IVD (Mosely et al., 2020). Here, we find that aging-related loss of IVD resistive force, which is consistent with loss of proteoglycan content (Holguin et al., 2014), was similarly reduced by OVX-induced estrogen depletion. Advanced aging also increases IVD collagen content (Holguin et al., 2014), which may have contributed to the age-related increases in loading stiffness and displacement determined here. By contrast, the use of hormone replacement therapy can increase IVD height in menopausal women compared with that in premenopausal and untreated menopausal women (Calleja-Agius et al., 2009). Similarly, we find that both aging and OVX reduced the expression of ER-α protein in the IVD. Consequently, both aging and OVX induced histological IVD degeneration and decreased IVD mechanical force, whereas injection of raloxifene stimulated ER-α protein expression, reduced the IVD degeneration score, and strengthened the IVD in both young and old female IVDs. Raloxifene and estrogen bind to ER-α and β-catenin (Goldstein et al., 2000) and promote the proliferation of NP and annulus fibrosus cells (Gruber et al., 2002). Further, estrogen-dependent expression of ER-α mediates 40% of the functional binding sites for forkhead box protein A2 (FoxA2) (Palierne et al., 2016), an essential transcription factor for the normal development of the NP. However, it is important to note that the severe degree of ER-α depletion by aging and OVX did not lead to similar detriments between aged and OVX IVD in histological score or mechanical force. Aging induced greater detriments than OVX to the IVD, suggesting that estrogen depletion is among other dysregulations in the induction of IVD degeneration. Raloxifene injection stimulated estrogen signaling in the IVD to promote extracellular matrix anabolism and IVD structure and strength in young and old female mice.

The mechanism by which raloxifene augmented IVD height may also include 2 non-estrogenic pathways. Although injection of raloxifene similarly enlarged IVD height in young-adult female and male mice, the upregulation of ER-α protein in male IVD was muted compared with that in female IVD. Similarly, raloxifene injection increased ER-α protein in old female mice but did not increase IVD height in old female mice. By contrast and similar to the pattern of regulation of IVD height, raloxifene injection upregulated the gene expression of Wnt signaling cotranscription factor β-catenin in both young-adult male and female IVD but did not do so in old female IVD. Therefore, the first non-estrogenic mechanism by which injection of raloxifene may promote IVD structure is potentiation of Wnt/β-catenin signaling (Kouzmenko et al., 2004; Jia et al., 2016). First, we previously found that stimulating Wnt signaling by genetic or pharmacological means increases IVD structure and mechanics (Kroon et al., 2022) and raloxifene may also be doing so here. Second, raloxifene may also function in a cell-independent manner to promote binding of incompressible, viscoelastic water to collagen (Gallant et al., 2014). Mechanically, injection of raloxifene increased the quasistatic (e.g., force and loading stiffness) and viscoelastic (energy dissipation and loss tangent) properties of the IVD in male and female mice. Therefore, a combination of ER-α, Wnt signaling, and disc hydration may be altering disc mechanics. While these data support that raloxifene has non-estrogenic mechanisms, the main mechanism of action of raloxifene is more likely to be stimulation of estrogen signaling. Nevertheless, there is still a need for further mechanistic studies to establish the pathways involved by the injection of raloxifene.

Postmenopausal women experience pain more frequently and at higher intensity than age-matched men (Vacca et al., 2014; Wang, 2015; Rosen et al., 2017). Women experience more low back pain after menopause than age-matched men (Wáng et al., 2016). However, whether there is a sexual dimorphism of pain-related behavior in mice is less clear. A retrospective study that compiled the physical behavior of 4,554 mice by open-field activity showed no difference between male and female mice (Fritz et al., 2017). By contrast, some studies show that female mice have more activity than males (Tucker and McCabe, 2017), whereas others show that females have more behavioral signs of pain than males (Vincent et al., 2019). SP is a nerve signaling neurotransmitter that has long been associated with IVD degeneration and discogenic pain (Freemont et al., 1997; Song et al., 2017). We found that ovariectomy and aging increased the expression of SP in IVD and bone highlighting the relation between estrogen and pain-related markers. In vertebrae, the number of osteocytes that expressed SP was greater in females than in males, whereas the gene expression of *Tac1* (gene precursor to SP) and *NGF* in the IVD was less in females than in males. These data suggest that the variable pain-related behavior by sex in mice may be associated with differential spine tissue expression of pain-related markers and highlight the importance of investigating the entire functional spinal unit and of biological sex in the interpretation of pain-related behavior.

Raloxifene may reduce back (Lyritis et al., 2010) and joint pain (Fujita et al., 2010) in postmenopausal women by reduction of SP in IVD and bone cells. Compared with men, women have less discogenic expression of ER-α protein, which may limit raloxifene-induced cell proliferation (Goldstein et al., 2000; Gruber et al., 2002), and both ER-α-depletion and aging can increase pain-related SP expression in the IVD (Song et al., 2017). We find that injection of raloxifene reduced the expression of pain-related marker SP in annulus fibrosus cells, gene precursor *Tac1* in the IVD, and SP in osteocytes of young-adult and old mice. We and others (Berman et al., 2016) found that raloxifene injection did not augment the bone quantity in the vertebral bone of male mice, but other skeletal sites may incur benefit. Our data indicate that injection of raloxifene in female and male mice reduced SP in the spine, but not all pain-related markers, as demonstrated by a lack of regulation of *NGF* gene expression in the IVD. Estrogen-deficiency further corroborated the role of estrogen in the regulation of SP by increasing the expression of SP in annulus fibrosus cells and osteocytes.

Despite several beneficial features of raloxifene in mice, our results require further investigation into the effect of raloxifene on aging male mice. We injected raloxifene in old female mice to demonstrate its efficacy in the current clinical population. It will be interesting to understand how raloxifene may influence behavioral assays such as gait movement and ambulatory velocity in mice. However, the decision to administer raloxifene for IVD degeneration and osteoporosis must be weighed against the greater incidence of stroke and venous thromboembolism (Mosca et al., 2009). Despite several beneficial features, our results implied that raloxifene had some surmountable physiological consequences and that our experimental approach may not have exposed the full translational potential of each therapeutic modality. First, raloxifene injection did not affect the tail bone structural properties or many tail IVD-related properties (data not shown). These data suggest that the lower mechanical loading environment of tail IVD than that of lumbar IVD may have limited the homogeneous distribution of raloxifene and its benefits (Silva and Holguin, 2020). Second, the tail may not be an ideal candidate to determine the consequences of aging on the spine. Here and in a previous study, we found that tail vertebrae of mice adapt differently to aging than lumbar vertebrae by increasing in structure and strength (Holguin et al., 2014). Lastly, a limitation that should be kept in mind is that CON mice did not receive PBS like aged control VEH mice, which is intended to control for factors associated with animal handling.

In conclusion, there is a great need for pharmacological therapies for IVD degeneration and the transition of regenerative therapies from bench to bedside includes several developmental stages that can take decades. Raloxifene has a couple of key advantages that can potentially expedite its use 1): It has been extensively screened for human safety and 2) is already applied to improve an influential tissue of the IVD—bone. Further study will be required to determine whether this therapeutic modality prevents severe IVD degeneration, and it will be interesting to see its effect on old male IVDs. Overall, systemic administration of raloxifene in young and old mice promotes IVD health and its implementation may limit the painful consequences of IVD degeneration in our lives. These findings suggest that the positive effects of commercially available raloxifene (Evista) to prevent osteoporosis can possibly be repurposed for retarding IVD degeneration and treating discogenic pain.

## Data Availability

The original contributions presented in the study are included in the article/Supplementary Material, further inquiries can be directed to the corresponding author.

## Supplementary Material

Supplementary Figure S1 Mechanical testing for dynamic loading. **(A, A’)** Micro-indentation system for mechanical loading. The IVD is isolated, submerged in PBS, and sinusoidally loaded. **(B)** Force–distance graph (hysteresis loop) starting at “1” and ending at “2” generated from the system.

Supplementary Figure S2 Negative controls of ER-α staining of **(A)** 4-month-old, **(B)** 24-month-old, and **(C)** OVX IVDs at moderate magnification. Scale: 100 μm.

Supplementary Figure S3 High magnification of the AF of (Left: **(A–C)**) ER-α–stained IVD and (Right: **(A–C)**) the respective negative controls. The presence of protein stain is indicated by an arrow, whereas the absence of protein staining is indicated by an arrowhead. Scale: 50 μm.

Supplementary Figure S4 High magnification of the NP of (Left: **(A–C)**) ER-α–stained IVD and (Right: **(A–C)**) the respective negative controls. The presence of protein stain is indicated by an arrow, whereas the absence of protein staining is indicated by an arrowhead. Scale: 50 μm.

Supplementary Figure S5 ER-α protein cell count in the AF was suppressed by OVX and elevated by raloxifene. Cell count of ER-α protein-expressing cells in the **(A)** AF and **(B)** NP of young-adult (4 months), OVX (6 months), and old (24 months) mice. Data are represented as box plots with mean marked as cross (x), 25/75% deviation lines, and maximum/minimum whiskers. *: Control (CON, *n* = 5/sex/group) vs raloxifene (RAL); R: CON vs RAL; S: male vs female; RxS: interaction; vehicle (VEH, *n* = 8/group) vs RAL, SHAM (*n* = 4–5/group) vs ovariectomized (OVX), *p* < 0.05. AF, annulus fibrosus; NP, nucleus pulposus; ND, not determined.

Supplementary Figure S6 Raloxifene improved the AF and NP structure of lumbar IVDs in young and old mice. 5x representative lumbar Safranin-O/Fast Green staining in young (4 months), OVX (6 months), and old (24 months) IVDs. AF, annulus fibrosus; and NP, nucleus pulposus. Scale: 100 μm.

Supplementary Figure S7 Raloxifene increased the NP area of lumbar IVDs in 4-month-old mice. (A) Nucleus pulposus area of young (4 months), OVX (6 months), and old (24 months) mice. Data are represented as box plots with mean marked as cross (x), 25/75% deviation lines, and maximum/minimum whiskers. *: Control (CON, *n* = 5/sex/group) vs raloxifene (RAL); R: CON vs RAL; S: male vs female; RxS: interaction; vehicle (VEH, *n* = 8/group) vs RAL, SHAM (*n* = 4–5/group) vs ovariectomized (OVX), *p* < 0.05.

Supplementary Figure S8 Negative controls of SP staining of **(A)** 4-month-old, **(B)** 24-month-old, and **(C)** OVX IVDs at moderate magnification. Scale: 100 μm.

Supplementary Figure S9 High magnification of the AF of (Left: **(A–C)**) SP-stained IVD and (Right-**(A–C)**) the respective negative controls. The presence of protein stain is indicated by an arrow, whereas the absence of protein staining is indicated by an arrowhead. Scale: 50 μm.

Supplementary Figure S10 High magnification of the NP of (Left: **(A–C)**) SP-stained IVD and (Right: **(A–C)**) the respective negative controls. The presence of protein stain is indicated by an arrow, whereas the absence of protein staining is indicated by an arrowhead. Scale: 50 μm.

Supplementary Figure S11 Substance P protein cell count in the AF was increased by OVX and suppressed by raloxifene. Cell count of substance protein expressing cells in the (A) AF and (B) NP of young-adult (4 months), OVX (6 months), and old (24 months) mice. Data are represented as box plots with mean marked as cross (x), 25/75% deviation lines, and maximum/minimum whiskers. *: Control (CON, *n* = 5/sex/group) vs. raloxifene (RAL); R: CON vs RAL; S: male vs. female; RxS: interaction; vehicle (VEH, *n* = 8/group) vs RAL, SHAM (*n* = 4–5/group) vs ovariectomized (OVX), p<0.05. AF, annulus fibrosus; NP: nucleus pulposus; and ND, not determined.

Supplementary Figure S12 ER-α staining in 4-month osteocytes. The presence of protein stain is indicated by an arrow, whereas the absence of protein staining is indicated by an arrowhead. Scale bar: 50 μm.

Supplementary Figure S13 Substance P staining in osteocytes at a **(A)** high magnification and the **(B)** negative control. The presence of protein stain is indicated by an arrow, whereas the absence of protein staining is indicated by an arrowhead. Scale bar: 50 μm.

## References

[B1] Baron Y. M. Brincat M. P. Galea R. Calleja N. (2005). Intervertebral Disc Height in Treated and Untreated Overweight Post-menopausal Women. Hum. Reprod. 20 (12), 3566–3570. 10.1093/humrep/dei251 16113041

[B2] Basso M. Cavagnaro L. Zanirato A. Divano S. Formica C. Formica M. (2017). What Is the Clinical Evidence on Regenerative Medicine in Intervertebral Disc Degeneration? Musculoskelet. Surg. 101 (2), 93–104. 10.1007/s12306-017-0462-3 28191592

[B3] Berman A. G. Wallace J. M. Bart Z. R. Allen M. R. (2016). Raloxifene Reduces Skeletal Fractures in an Animal Model of Osteogenesis Imperfecta. Matrix Biol. 52-54, 19–28. 10.1016/j.matbio.2015.12.008 26707242

[B4] Bivi N. Hu H. Chavali B. Chalmers M. J. Reutter C. T. Durst G. L. (2016). Structural Features Underlying Raloxifene's Biophysical Interaction with Bone Matrix. Bioorg. Med. Chem. 24 (4), 759–767. 10.1016/j.bmc.2015.12.045 26795112

[B5] Calleja-Agius J. Muscat-Baron Y. Brincat M. P. (2009). Estrogens and the Intervertebral Disc. Menopause Int. 15 (3), 127–130. 10.1258/mi.2009.009016 19723683

[B6] Caprez S. Menzel U. Li Z. Grad S. Alini M. Peroglio M. (2018). Isolation of High‐quality RNA from Intervertebral Disc Tissue via Pronase Predigestion and Tissue Pulverization. JOR Spine 1 (2), e1017. 10.1002/jsp2.1017 31463444 PMC6686795

[B7] Dahia C. L. Mahoney E. J. Durrani A. A. Wylie C. (2009). Intercellular Signaling Pathways Active during Intervertebral Disc Growth, Differentiation, and Aging. Spine (Phila Pa 1976) 34, 456–462. 10.1097/BRS.0b013e3181913e98 19212276

[B8] De Schepper E. I. Damen J. van Meurs J. B. Ginai A. Z. Popham M. Hofman A. (2010). The Association between Lumbar Disc Degeneration and Low Back Pain: The Influence of Age, Gender, and Individual Radiographic Features. Spine (Phila Pa 1976) 35 (5), 531–536. 10.1097/BRS.0b013e3181aa5b33 20147869

[B9] Dowdell J. Erwin M. Choma T. Vaccaro A. Iatridis J. Cho S. K. (2017). Intervertebral Disk Degeneration and Repair. Neurosurgery 80 (3), S46–S54. 10.1093/neuros/nyw078 28350945 PMC5585783

[B10] Ettinger B. Mitlalc B. H. Nickelsen T. Genant H. K. Christiansen C. Zanchetta J. R. (1999). Reduction of Vertebral Fracture Risk in Postmenopausal Women with Osteoporosis Treated with RaloxifeneResults from a 3-Year Randomized Clinical Trial. J. Am. Med. Assoc. 282 (7), 637–645. 10.1001/jama.282.7.637 10517716

[B11] Freemont A. Peacock T. Goupille P. Hoyland J. O'Brien J. Jayson M. (1997). Nerve Ingrowth into Diseased Intervertebral Disc in Chronic Back Pain. Lancet 350 (9072), 178–181. 10.1016/s0140-6736(97)02135-1 9250186

[B12] Fritz A.-K. Amrein I. Wolfer D. P. (2017). Similar Reliability and Equivalent Performance of Female and Male Mice in the Open Field and Water-Maze Place Navigation Task. Am. J. Med. Genet. 175 (3), 380–391. 10.1002/ajmg.c.31565 28654717 PMC5638061

[B13] Fuchs-Young R. Glasebrook A. l. Short L. l. Draper M. w. Rippy M. k. Cole H. w. (1995). Raloxifene Is a Tissue-Selective Agonist/antagonist that Functions through the Estrogen Receptor. Ann. N. Y. Acad. Sci. 761 (1), 355–360. 10.1111/j.1749-6632.1995.tb31392.x 7625735

[B14] Fujita T. Fujii Y. Munezane H. Ohue M. Takagi Y. (2010). Analgesic Effect of Raloxifene on Back and Knee Pain in Postmenopausal Women with Osteoporosis And/or Osteoarthritis. J. Bone Min. Metab. 28 (4), 477–484. 10.1007/s00774-009-0155-6 20157745

[B15] Gallant M. A. Brown D. M. Hammond M. Wallace J. M. Du J. Almer J. D. (2014). Bone Cell-independent Benefits of Raloxifene on the Skeleton: A Novel Mechanism for Improving Bone Material Properties. Bone 61, 191–200. 10.1016/j.bone.2014.01.009 24468719 PMC3955028

[B16] Gambacciani M. Pepe A. Cappagli B. Palmieri E. Genazzani A. R. (2007). The Relative Contributions of Menopause and Aging to Postmenopausal Reduction in Intervertebral Disk Height. Climacteric 10 (4), 298–305. 10.1080/13697130701457729 17653956

[B17] Goldstein S. R. Siddhanti S. Ciaccia A. V. Plouffe L. (2000). A Pharmacological Review of Selective Oestrogen Receptor Modulators. Hum. Reprod. Update 6 (3), 212–224. 10.1093/humupd/6.3.212 10874566

[B18] Gruber H. E. Yamaguchi D. Ingram J. Leslie K. Huang W. Miller T. A. (2002). Expression and Localization of Estrogen Receptor-Beta in Annulus Cells of the Human Intervertebral Disc and the Mitogenic Effect of 17-Beta-Estradiol *In Vitro* . BMC Musculoskelet. Disord. 3, 4–5. 10.1186/1471-2474-3-4 11846890 PMC65546

[B19] Gullbrand S. E. Smith L. J. Smith H. E. Mauck R. L. (2018). Promise, Progress, and Problems in Whole Disc Tissue Engineering. Jor Spine 1 (2), e1015. 10.1002/jsp2.1015 31463442 PMC6686799

[B20] Gullbrand S. E. Ashinsky B. G. Bonnevie E. D. Kim D. H. Engiles J. B. Smith L. J. (2018). Long-term Mechanical Function and Integration of an Implanted Tissue-Engineered Intervertebral Disc. Sci. Transl. Med. 10 (468), 1–11. 10.1126/scitranslmed.aau0670 PMC7380504 30463917

[B21] Holguin N. Silva M. J. (2018). *In-Vivo* Nucleus Pulposus-Specific Regulation of Adult Murine Intervertebral Disc Degeneration via Wnt/Beta-Catenin Signaling. Sci. Rep. 8 (1), 11191–11214. 10.1038/s41598-018-29352-3 30046041 PMC6060169

[B22] Holguin N. Muir J. Rubin C. Judex S. (2009). Short Applications of Very Low-Magnitude Vibrations Attenuate Expansion of the Intervertebral Disc during Extended Bed Rest. Spine J. 9 (6), 470–477. 10.1016/j.spinee.2009.02.009 19356986

[B23] Holguin N. Uzer G. Chiang F.-P. Rubin C. Judex S. (2011). Brief Daily Exposure to Low-Intensity Vibration Mitigates the Degradation of the Intervertebral Disc in a Frequency-specific Manner. J. Appl. Physiol. 111 (6), 1846–1853. 10.1152/japplphysiol.00846.2011 21960658 PMC3233878

[B24] Holguin N. Martin J. T. Elliott D. M. Judex S. (2013). Low-intensity Vibrations Partially Maintain Intervertebral Disc Mechanics and Spinal Muscle Area during Deconditioning. Spine J. 13 (4), 428–436. 10.1016/j.spinee.2013.01.046 23507530 PMC3628078

[B25] Holguin N. Aguilar R. Harland R. A. Bomar B. A. Silva M. J. (2014). The Aging Mouse Partially Models the Aging Human Spine: Lumbar and Coccygeal Disc Height, Composition, Mechanical Properties, and Wnt Signaling in Young and Old Mice. J. Appl. Physiol. 116 (12), 1551–1560. 10.1152/japplphysiol.01322.2013 24790018 PMC4064379

[B26] Homminga J. Aquarius R. Bulsink V. E. Jansen C. T. J. Verdonschot N. (2012). Can Vertebral Density Changes Be Explained by Intervertebral Disc Degeneration? Med. Eng. Phys. 34 (4), 453–458. 10.1016/j.medengphy.2011.08.003 21893424

[B27] Hoy D. Bain C. Williams G. March L. Brooks P. Blyth F. (2012). A Systematic Review of the Global Prevalence of Low Back Pain. Arthritis Rheum. 64 (6), 2028–2037. 10.1002/art.34347 22231424

[B28] Jia H. Ma J. Lv J. Ma X. Xu W. Yang Y. (2016). Oestrogen and Parathyroid Hormone Alleviate Lumbar Intervertebral Disc Degeneration in Ovariectomized Rats and Enhance Wnt/β-Catenin Pathway Activity. Sci. Rep. 6 (22), 27521. 10.1038/srep27521 27279629 PMC4899752

[B39] Katz J. N. (2006). Lumbar Disc Disorders and Low-Back Pain: Socioeconomic Factors and Consequences. J. Bone Joint Surg. 88 (2), 21–24. 10.2106/JBJS.E.01273 16595438

[B29] Kouzmenko A. P. Takeyama K.-i. Ito S. Furutani T. Sawatsubashi S. Maki A. (2004). Wnt/β-Catenin and Estrogen Signaling Converge *In Vivo* . J. Biol. Chem. 279 (39), 40255–40258. 10.1074/jbc.c400331200 15304487

[B30] Kroon T. Bhadouria N. Niziolek P. Edwards D. Choi R. Clinkenbeard E. L. (2022). Suppression of Sost/Sclerostin and Dickkopf‐1 Augment Intervertebral Disc Structure in Mice. J Bone Miner. Res. 37, 1156–1169. 10.1002/jbmr.4546 35278242 PMC9320845

[B31] Liu J. W. Abraham A. C. Y. Tang S. (2015). The High-Throughput Phenotyping of the Viscoelastic Behavior of Whole Mouse Intervertebral Discs Using a Novel Method of Dynamic Mechanical Testing. J. Biomech. 48 (10), 2189–2194. 10.1016/j.jbiomech.2015.04.040 26004435 PMC4492880

[B32] Livshits G. Ermakov S. Popham M. MacGregor A. J. Sambrook P. N. Spector T. D. (2010). Evidence that Bone Mineral Density Plays a Role in Degenerative Disc Disease: The UK Twin Spine Study. Ann. Rheum. Dis. 69 (12), 2102–2106. 10.1136/ard.2010.131441 20570838 PMC3002767

[B33] Loibl M. Wuertz‐Kozak K. Vadala G. Lang S. Fairbank J. Urban J. P. (2019). Controversies in Regenerative Medicine: Should Intervertebral Disc Degeneration Be Treated with Mesenchymal Stem Cells? Jor Spine 2 (1), e1043. 10.1002/jsp2.1043 31463457 PMC6711491

[B34] Lyritis G. Marin F. Barker C. Pfeifer M. Farrerons J. Brixen K. (2010). Back Pain during Different Sequential Treatment Regimens of Teriparatide: Results from Eurofors. Curr. Med. Res. Opin. 26 (8), 1799–1807. 10.1185/03007995.2010.488516 20482322

[B35] McCann M. Séguin C. (2016). Notochord Cells in Intervertebral Disc Development and Degeneration. J. Dev. Biol. 4 (1), 3. 10.3390/jdb4010003 27252900 PMC4885739

[B36] Messalli E. Scaffa C. (2009). Long-Term Safety and Efficacy of Raloxifene in the Prevention and Treatment of Postmenopausal Osteoporosis: An Update. Int. J. Womens Health 1 (1), 11–20. 10.2147/ijwh.s3894 PMC2971719 21072271

[B37] Mosca L. Grady D. Barrett-Connor E. Collins P. Wenger N. Abramson B. L. (2009). Effect of Raloxifene on Stroke and Venous Thromboembolism According to Subgroups in Postmenopausal Women at Increased Risk of Coronary Heart Disease. Stroke 40 (1), 147–155. 10.1161/strokeaha.108.518621 18948611 PMC3559135

[B38] Mosely G. E. Wang M. Nassar P. Lai A. Charen D. A. Zhang B. (2020). Males and Females Exhibit Distinct Relationships between Intervertebral Disc Degeneration and Pain in a Rat Model. Sci. Rep. 10, 15120. 10.1038/s41598-020-72081-9 32934258 PMC7492468

[B40] Palierne G. Fabre A. Solinhac R. Le Péron C. Avner S. Lenfant F. (2016). Changes in Gene Expression and Estrogen Receptor Cistrome in Mouse Liver upon Acute E2 Treatment. Mol. Endocrinol. 30 (7), 709–732. 10.1210/me.2015-1311 27164166 PMC5426578

[B41] Papadokostakis G. Katonis P. Damilakis J. Hadjipavlou A. (2005). Does Raloxifene Treatment Influence Back Pain and Disability Among Postmenopausal Women with Osteoporosis? Eur. Spine J. 14 (10), 977–981. 10.1007/s00586-005-0899-1 15834592

[B42] Risbud M. V. Schaer T. P. Shapiro I. M. (2010). Toward an Understanding of the Role of Notochordal Cells in the Adult Intervertebral Disc: From Discord to Accord. Dev. Dyn. 239 (8), 2141–2148. 10.1002/dvdy.22350 20568241 PMC3634351

[B43] Roberts S. Evans H. Trivedi J. Menage J. (2006). Histology and Pathology of the Human Intervertebral Disc. J. Bone Jt. Surg.-Am. Vol. 88 (Suppl. 2), 10–14. 10.2106/00004623-200604002-00003 16595436

[B44] Rosen S. Ham B. Mogil J. S. (2017). Sex Differences in Neuroimmunity and Pain. J. Neurosci. Res. 95 (1–2), 500–508. 10.1002/jnr.23831 27870397

[B45] Silva M. J. Holguin N. (2020). Aging Aggravates Intervertebral Disc Degeneration by Regulating Transcription Factors toward Chondrogenesis. FASEB J. 34 (2), 1970–1982. 10.1096/fj.201902109r 31909538 PMC7018543

[B46] Song X.-X. Shi S. Guo Z. Li X.-F. Yu B.-W. (2017). Estrogen Receptors Involvement in Intervertebral Discogenic Pain of the Elderly Women: Colocalization and Correlation with the Expression of Substance P in Nucleus Pulposus. Oncotarget 8 (24), 38136–38144. 10.18632/oncotarget.15421 28430617 PMC5503520

[B47] Tam V. Chan W. C. W. Leung V. Y. L. Cheah K. S. E. Cheung K. M. C. Sakai D. (2018). Histological and Reference System for the Analysis of Mouse Intervertebral Disc. J. Orthop. Res. 36 (1), 233–243. 10.1002/jor.23637 28636254

[B48] Taranta A. Brama M. Teti A. De luca V. Scandurra R. Spera G. (2002). The Selective Estrogen Receptor Modulator Raloxifene Regulates Osteoclast and Osteoblast Activity *In Vitro* . Bone 30 (2), 368–376. 10.1016/s8756-3282(01)00685-8 11856644

[B49] Tucker L. B. McCabe J. T. (2017). Behavior of Male and Female C57Bl/6J Mice Is More Consistent with Repeated Trials in the Elevated Zero Maze Than in the Elevated Plus Maze. Front. Behav. Neurosci. 11 (13), 1–8. 10.3389/fnbeh.2017.00013 28184191 PMC5266707

[B50] Vacca V. Marinelli S. Pieroni L. Urbani A. Luvisetto S. Pavone F. (2014). Higher Pain Perception and Lack of Recovery from Neuropathic Pain in Females: A Behavioural, Immunohistochemical, and Proteomic Investigation on Sex-Related Differences in Mice. Pain 155 (2), 388–402. 10.1016/j.pain.2013.10.027 24231652

[B51] Vasiliadis E. S. Pneumaticos S. G. Evangelopoulos D. S. Papavassiliou A. G. (2014). Biologic Treatment of Mild and Moderate Intervertebral Disc Degeneration. Mol. Med. 20 (1), 400–409. 10.2119/molmed.2014.00145 25171110 PMC4212014

[B52] Vincent K. Mohanty S. Pinelli R. Bonavita R. Pricop P. Albert T. J. (2019). Aging of Mouse Intervertebral Disc and Association with Back Pain. Bone 123, 246–259. 10.1016/j.bone.2019.03.037 30936040 PMC6549718

[B53] Wáng Y. X. J. Wáng J. Q. Káplár Z. (2016). Increased Low Back Pain Prevalence in Females Than in Males after Menopause Age: Evidences Based on Synthetic Literature Review. Quant. Imaging Med. Surg. 6 (2), 199–206. 10.21037/qims.2016.04.06 27190772 PMC4858456

[B54] Wang Y. X. J. (2015). Postmenopausal Chinese Women Show Accelerated Lumbar Disc Degeneration Compared with Chinese Men. J. Orthop. Transl. 3 (4), 205–211. 10.1016/j.jot.2015.09.001 PMC5986995 30035059

[B55] Zhao C.-Q. Wang L.-M. Jiang L.-S. Dai L.-Y. (2007). The Cell Biology of Intervertebral Disc Aging and Degeneration. Ageing Res. Rev. 6 (3), 247–261. 10.1016/j.arr.2007.08.001 17870673

